# Supplementary material for: Comparative analysis of drug-salt-polymer interactions by experiment and molecular simulation improves biopharmaceutical performance
Source: Commun Chem. 2023 Sep 25;6:201. doi: 10.1038/s42004-023-01006-0 (PMC10519957; doi:10.1038/s42004-023-01006-0)
Supplement: Supplementary file 2 — Description of Additional Supplementary Files [file 42004_2023_1006_MOESM2_ESM.pdf]

Description of Additional Supplementary Files

File name: Supplementary Data 1

Description: FTIR and Raman Data for Fig. 2a-d.

File name: Supplementary Data 2

Description: Solubility, Dissolution & Pharmacokinetic Data for Fig. 6a-c.

File name: Supplementary Data 3

Description: Powder X-Ray Diffractogram Data for Fig. S5a
