# Supplementary material for: Comparative analysis of drug-salt-polymer interactions by experiment and molecular simulation improves biopharmaceutical performance
Source: Commun Chem. 2023 Sep 25;6:201. doi: 10.1038/s42004-023-01006-0 (PMC10519957; doi:10.1038/s42004-023-01006-0)
Supplement: Supplementary file 6 — Reporting Summary [file 42004_2023_1006_MOESM6_ESM.pdf]

nature portfolio

Corresponding author(s): Sangamwar, Wade

Last updated by author(s): 09/09/2023

Reporting Summary

Nature Portfolio wishes to improve the reproducibility of the work that we publish. This form provides structure for consistency and transparency in reporting. For further information on Nature Portfolio policies, see our Editorial Policies and the Editorial Policy Checklist.

Statistics

For all statistical analyses, confirm that the following items are present in the figure legend, table legend, main text, or Methods section.

n/a	Confirmed	
☐	☒	The exact sample size (*n*) for each experimental group/condition, given as a discrete number and unit of measurement
☐	☒	A statement on whether measurements were taken from distinct samples or whether the same sample was measured repeatedly
☐	☒	The statistical test(s) used AND whether they are one- or two-sided<br>*Only common tests should be described solely by name; describe more complex techniques in the Methods section.*
☐	☒	A description of all covariates tested
☐	☒	A description of any assumptions or corrections, such as tests of normality and adjustment for multiple comparisons
☐	☒	A full description of the statistical parameters including central tendency (e.g. means) or other basic estimates (e.g. regression coefficient) AND variation (e.g. standard deviation) or associated estimates of uncertainty (e.g. confidence intervals)
☐	☒	For null hypothesis testing, the test statistic (e.g. *F*, *t*, *r*) with confidence intervals, effect sizes, degrees of freedom and *P* value noted<br>*Give P values as exact values whenever suitable.*
☒	☐	For Bayesian analysis, information on the choice of priors and Markov chain Monte Carlo settings
☒	☐	For hierarchical and complex designs, identification of the appropriate level for tests and full reporting of outcomes
☒	☐	Estimates of effect sizes (e.g. Cohen's *d*, Pearson's *r*), indicating how they were calculated

Our web collection on statistics for biologists contains articles on many of the points above.

Software and code

Policy information about availability of computer code

Data collection	Software for all molecular modeling and simulation is described in methods and is available (Schrödinger software, AMBER versions 14 and 20, Gaussian 09 software, Gromacs). All scripts for running and analysing simulations are available in the software packages used or in the zenodo repository at: https://doi.org/10.5281/zenodo.8228709.
Data analysis	Molecular smulation results were analysed and visualized using the above software tools or XMgrace, pymol or vmd, all available as described in methods. Analysis of experimental data was performed with GraphPad Prism 9 software, version 9.1.0 (221) (GraphPad Software, LLC. San Diego, USA) .

For manuscripts utilizing custom algorithms or software that are central to the research but not yet described in published literature, software must be made available to editors and reviewers. We strongly encourage code deposition in a community repository (e.g. GitHub). See the Nature Portfolio guidelines for submitting code & software for further information.

Data

Policy information about availability of data

All manuscripts must include a data availability statement. This statement should provide the following information, where applicable:

- Accession codes, unique identifiers, or web links for publicly available datasets
- A description of any restrictions on data availability
- For clinical datasets or third party data, please ensure that the statement adheres to our policy

All data are available in the main text or the supplementary information and data files or the repository at zenodo at: https://doi.org/10.5281/zenodo.8228709. Materials are available from the authors on reasonable request.

Human research participants

Policy information about studies involving human research participants and Sex and Gender in Research.

Reporting on sex and gender	No human research
Population characteristics	*Describe the covariate-relevant population characteristics of the human research participants (e.g. age, genotypic information, past and current diagnosis and treatment categories). If you filled out the behavioural & social sciences study design questions and have nothing to add here, write "See above."*
Recruitment	*Describe how participants were recruited. Outline any potential self-selection bias or other biases that may be present and how these are likely to impact results.*
Ethics oversight	*Identify the organization(s) that approved the study protocol.*

Note that full information on the approval of the study protocol must also be provided in the manuscript.

Field-specific reporting

Please select the one below that is the best fit for your research. If you are not sure, read the appropriate sections before making your selection.

☒ Life sciences ☐ Behavioural & social sciences ☐ Ecological, evolutionary & environmental sciences

For a reference copy of the document with all sections, see nature.com/documents/nr-reporting-summary-flat.pdf

Life sciences study design

All studies must disclose on these points even when the disclosure is negative.

Sample size	No statistical method or sample-size calculation was applied to predetermine sample size. The sample size for pharmacokinetic (PK) studies was chosen according to established methods in animal studies research papers and recommendations of animal research committees. The sample size chosen was 5 animals, each from one of 7 groups as per the availability of animals for lab scale research and the approved animal protocol (IAEC/19/51-R). This sample size was chosen as the smallest to avoid unnecessary waste of resources and ethical issues while being large enough to identify any deviating data (outliers) and produce repeatability to validate/verify the results. The in vitro studies were performed in triplicate which was sufficient to assess the study outcomes on a lab scale.
Data exclusions	The pharmacokinetic (PK) experiments were performed in 5 individual animals, each from one of 7 groups. The results were reproducible but there were one or two outliers in the data points at different time points. To avoid misleading average values, we excluded these outliers. The exclusion criteria for the outliers were not pre-established but were based on analysis of the measured data points. Outliers in preclinical PK studies are commonly observed, due to manual variability. The exclusion of the outliers did not affect the overall inference from the PK studies on the bioavailability of the new formations in comparison to the crystalline drug. Average values were computed after removal of outliers and statistical data analysis was done by one-way analysis of variance (ANOVA) by applying the Bonferroni test for multiple comparison of dependent variables. The results were considered statistically significant when $p < 0.05$.
Replication	The in vitro experiments were performed in triplicate and, the in vivo PK studies were done 5 times using 5 animals in each group as per approved animal protocol (IAEC/19/51-R) to test the repeatability of the experiments. This resulted in reproducibility of data as shown in the manuscript and the measured PK data points, which are provided in the accompanying Excel sheet. All replication attempts were successful, however, during data analysis for the PK studies, we observed some outliers at different time points for different groups. Despite performing experiments in animals of the same species and same body weight and age under the same environmental conditions, there were outliers, which could be due to manual errors viz. sampling error while sample collection, processing of samples or dilution error.
Randomization	For the PK studies, female SD rats of the same age, species and strain were allocated randomly into 7 groups each containing pre-defined numbers (5 rats) in the weight range of 200 ± 30 g and measurements were performed for 5 rats, each taken from a different group.

Randomisation was done to nullify the variations (known and unknown) that could bias the results. Different treatment groups were processed under the same conditions and measurements were taken at the same time intervals. All the groups were habituated under the same environmental conditions (12 h light-dark cycles at 25 °C and 60 % RH) with food and were fasted for 12 h with free access to water before the start of experiments. The same conditions were maintained during the whole process from dosing of animals to sample collection, processing and analysing of samples to eliminate all types of variations.

Blinding

The investigators were not blinded to group allocation during data collection and analysis as it is not possible or relevant for the PK studies. In the PK studies, we assessed the bioavailability of celecoxib (CEL) and its different formulations. The investigator needed to know the identity of the allocated group as dosing in animals was done based on the drug loading in the formulation and the recommended dose of the drug which was calculated and administered according to 10 mg equivalent of CEL/kg of rat body mass. Furthermore, in order to compare the bioavailability of CEL and its amorphous formulations, groups were allocated and labelled after random distribution to avoid any misinterpretation of results.

Reporting for specific materials, systems and methods

We require information from authors about some types of materials, experimental systems and methods used in many studies. Here, indicate whether each material, system or method listed is relevant to your study. If you are not sure if a list item applies to your research, read the appropriate section before selecting a response.

Materials & experimental systems

n/a	Involved in the study
☒	☐ Antibodies
☒	☐ Eukaryotic cell lines
☒	☐ Palaeontology and archaeology
☒	☐ Animals and other organisms
☒	☐ Clinical data
☒	☐ Dual use research of concern

Methods

n/a	Involved in the study
☒	☐ ChIP-seq
☒	☐ Flow cytometry
☒	☐ MRI-based neuroimaging
